# Perovskite‐Gallium Nitride Tandem Light‐Emitting Diodes with Improved Luminance and Color Tunability

**DOI:** 10.1002/advs.202201844

**Published:** 2022-05-21

**Authors:** Zong‐Tao Li, Hong‐Wei Zhang, Jia‐Sheng Li, Kai Cao, Ziming Chen, Liang Xu, Xin‐Rui Ding, Bin‐Hai Yu, Yong Tang, Jian‐Zhen Ou, Hao‐Chung Kuo, Hin‐Lap Yip

**Affiliations:** ^1^ National and Local Joint Engineering Research Center of Semiconductor Display and Optical Communication Devices South China University of Technology Guangzhou 510641 China; ^2^ Department of Chemistry Imperial College London London W12 0BZ United Kingdom; ^3^ R&D Center Foshan Nationstar Semiconductor Technology Co. Ltd. Foshan 528000 China; ^4^ School of Engineering RMIT University Melbourne Victoria 3000 Australia; ^5^ Department of Photonics and Institute of Electro‐Optical Engineering College of Electrical and Computer Engineering National Chiao Tung University Hsinchu 30010 Taiwan, China; ^6^ Semiconductor Research Center Hon Hai Research Institute New Taipei City Taiwan 236 China; ^7^ Department of Materials Science and Engineering City University of Hong Kong Hong Kong 999077 China; ^8^ School of Energy and Environment City University of Hong Kong Hong Kong 999077 China; ^9^ Hong Kong Institute for Clean Energy City University of Hong Kong Hong Kong 999077 China

**Keywords:** gallium nitride light‐emitting diodes, luminance and color tunability, perovskite light‐emitting diodes, tandem structure

## Abstract

Tandem structures with different subpixels are promising for perovskite‐based multicolor electroluminescence (EL) devices in ultra‐high‐resolution full‐color displays; however, realizing excellent luminance‐ and color‐independent tunability considering the low brightness and stability of blue perovskite light‐emitting diodes (PeLEDs) remains a challenge. Herein, a bright and stable blue gallium nitride (GaN) LED is utilized for vertical integration with a green MAPbBr_3_ PeLED, successfully achieving a Pe‐GaN tandem LED with independently tunable luminance and color. The electronic and photonic co‐excitation (EPCE) effect is found to suppress the radiative recombination and current injection of PeLEDs, leading to degraded luminance and current efficiency under direct current modulation. Accordingly, the pulse‐width modulation is introduced to the tandem device with a negligible EPCE effect, and the average hybrid current efficiency is significantly improved by 139.5%, finally achieving a record tunable luminance (average tuning range of 16631 cd m^−2^ at an arbitrary color from blue to green) for perovskite‐based multi‐color LEDs. The reported excellent independent tunability can be the starting point for perovskite‐based multicolor EL devices, enabling the combination with matured semiconductor technologies to facilitate their commercialization in advanced display applications with ultra‐high resolution.

## Graphical abstract

A novel tandem structure is developed by depositing a green PeLED on a blue GaNLED substrate, where the indium–tin oxide serves as the co‐anode, enabling independent control of luminance and color for perovskite‐GaN LEDs. The tandem LED achieves a high average maximum hybrid luminance of 16631 cd m^−2^ at an arbitrary color from pure blue to green.

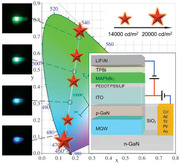

## 1 Introduction

Perovskite light‐emitting diodes (PeLEDs) have attracted significant attention because of their unique optoelectronic properties and low‐cost solution processability.^[^ ^1^, ^2^, ^3^, ^4^, ^5^, ^6^, ^7^ ^]^ Since the first demonstration of room‐temperature 3D PeLEDs in 2014,^[^ ^8^ ^]^ substantial progress has been achieved regarding monochromatic PeLEDs^[^ ^9^, ^10^, ^11^, ^12^ ^]^ with maximum external quantum efficiencies (EQEs) achieving 23%,^[^ ^13^ ^]^ 28%,^[^ ^14^ ^]^ and 12.3%^[^ ^15^ ^]^ for red, green, and blue emissions, respectively. Moreover, the sharp emission spectra of PeLEDs contribute to excellent color purity. Both the high device efficiency and sharp emission spectra make PeLEDs a potential candidate for full‐color display applications with a wide color gamut.^[^ ^16^ ^]^ However, the successful integration of red, green, and blue PeLEDs for multicolor electroluminescence (EL) pixels remains a significant challenge.

In conventional display panels, the red, green, and blue sub‐pixels are assembled in a side‐by‐side structure,^[^ ^17^, ^18^, ^19^ ^]^ which intrinsically leads to a large pixel area, making it difficult to fulfill the requirements of applications requiring ultra‐high display resolution, for example, advanced high‐dynamic‐range and virtual‐reality displays.^[^ ^20^, ^21^ ^]^ One efficient approach to reduce the pixel area is to combine two (or even three) colors (red, green, or blue) within one sub‐pixel; however, this requires excellent color tunability for the lighting device. To meet this demand, multicolor EL devices with a tandem structure are becoming a straightforward solution to improve space utilization.^[^ ^22^, ^23^ ^]^ In the PeLED field, the series connection is a typical tandem structure that integrates different emission colors. For example, Choy et al.^[^ ^24^ ^]^ reported a tandem PeLED with simultaneous emission from blue and red perovskite emitters. However, as this tandem device was based on series connection and driven by only a single power source, the ratio between these two emissions could not be independently controlled to realize the required color tunability. In addition to the series connection, a parallel connection achieved by introducing an intermediate connecting electrode (ICE) and therefore able to be driven by two power sources, has been extensively explored in organic and quantum‐dot tandem LEDs to independently control each sub‐color.^[^ ^25^, ^26^, ^27^ ^]^ This strategy has demonstrated significant potential for achieving excellent luminance and color tunability, which has been rarely reported in perovskite‐based multicolor EL devices. However, achieving a parallel‐based tandem device fully based on PeLEDs must address two main challenges: i) the inferior luminance and spectrum stability of blue (especially deep‐blue) PeLEDs limits the effective color control of a multicolor EL device;^[^ ^28^, ^29^ ^]^ ii) if no appropriate protection is applied, the bottom blue perovskite emitter could be negatively influenced when fabricating the upper functional layers owing to the low physical and chemical resistance of soft perovskites.^[^ ^30^, ^31^, ^32^ ^]^

In this study, to eliminate the weakness of the bottom blue perovskite emitter in a parallel‐based tandem device, a commercial blue gallium nitride (GaN) LED with excellent brightness and stability is used to vertically integrate a green MAPbBr_3_ PeLED by introducing indium tin oxide (ITO) as an ICE. Such a Pe‐GaN tandem LED can be driven by two individual power sources; hence, the luminance and color of the device can be controlled independently. Most importantly, we found that when using direct current modulation (DC mode), the electronic and photonic co‐excitation (EPCE) effect occurs within the green PeLED, which degrades the device performance. To minimize such a negative effect, we further introduce pulse‐width modulation (PWM) mode to significantly improve the device efficiency. Accordingly, we obtain a powerful Pe‐GaN tandem LED with precise color control from pure blue to green with an average luminance of 16 631 cd m^−2^, which is the record tunable luminance for perovskite‐based multi‐color LEDs. This work realizes excellent luminance‐ and color‐independent tunability of perovskite‐based multicolor EL devices for the first time, enabling their combination with matured semiconductor technologies to achieve advanced display applications with ultra‐high resolution.

## 2 Results and Discussion

### 2.1 Device Structure

Blue GaNLEDs have achieved significant success in commercialization because of their excellent stability, color purity, and brightness,^[^ ^33^, ^34^ ^]^ which were used as bottom LEDs in our study and prepared following the standard commercial process with a patterned sapphire substrate (PSS); see image in Figure S1, Supporting Information. The EL emission of GaNLEDs has an emission peak at 456 nm and a narrow full width at half of ≈20 nm (Figure S2a, Supporting Information), exhibiting a high‐purity blue color. Moreover, GaNLEDs exhibit high luminance of greater than 24 000 cd m^−2^ at relatively low voltages (Figure S2b, Supporting Information), surpassing the majority of the blue PeLEDs reported to date. Note that the current density of the proposed GaNLEDs increases sharply with increasing voltage (Figure S2c, Supporting Information), which also leads to a relatively low maximum current efficiency (CE) of 4.7 cd A^−1^ (Figure S2d, Supporting Information). These results can be attributed to the poor hole injection of the proposed GaNLEDs due to the large hole injection barrier, which is caused by the non‐ohmic contact.^[^ ^34^ ^]^ Nevertheless, the brightness of the blue GaNLEDs is high enough to adjust the emission color of tandem devices over a wide range of luminance.

**Figure 1:**
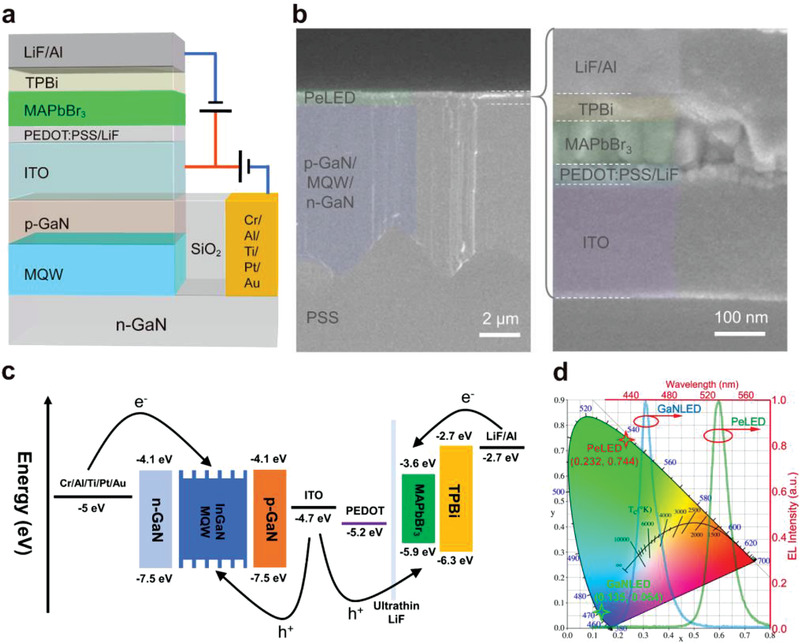
Device structure of Pe‐GaN tandem LEDs. a) Structure diagram with different driving modes. b) Cross‐sectional scanning electron microscope images. c) Energy level alignment. d) Color coordinates (CIE‐1931) and EL spectra of GaNLED and PeLED units.

Because of the excellent physical and chemical stability of GaNLEDs, they can also serve as a substrate for solution‐processing green PeLED units to obtain a Pe‐GaN tandem LED. The structure of the proposed tandem LEDs is displayed in **Figure** **1**a, where the GaNLED and PeLED units are connected by an ITO‐ICE as the co‐anode, where Cr/Al/Ti/Pt/Au and LiF/Al are the individual cathodes for the GaNLED and PeLED, respectively. The cross‐sectional image of the device displayed in Figure 1b confirms the vertical integration of the GaNLED and PeLED units, where the smooth epitaxial surface of the p‐GaN also facilitates the upper solution‐processed perovskite layer. This device structure has a parallel connection and can be driven by two different power sources separately (i.e., holes are injected from the ITO‐ICE co‐anode and electrons are injected from the individual metal cathode of each unit, as indicated in Figure 1c, to independently control the EL process). It should be noted that GaNLED and PeLED units have pure blue (0.135, 0.064) and green (0.232, 0.744) color coordinates (CIE‐1931), respectively (Figure 1d), which can satisfy the display demand on a wide‐color gamut after realizing the luminance‐ and color‐independent tunability.

### 2.2 Color Tunability under DC Mode

**Figure 2:**
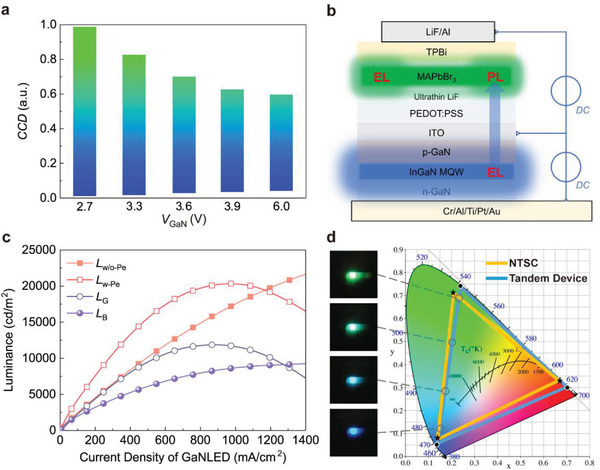
Color tunability of Pe‐GaN tandem LEDs under DC mode. a) CCD and normalized EL spectra of tandem devices with different initial *V*_GaN_, where the CCD is regulated by scanning the voltage of PeLED units. b) Illustration of the EPCE process. c) Luminance of GaNLED units with and without PeLED. d) Color gamut of tandem devices combined with a common red emitter.

Considering that the DC mode is the most general and simplest method to drive an LED device, the color tunability of tandem devices was first studied in the DC mode by fixing the voltage of the GaNLED (*V*_GaN_) to a certain value and scanning voltage of the PeLED (*V*_Pe_) from 3 to 11 V. To simplify the discussion of the color tunability of Pe‐GaN tandem LEDs under different driving conditions, the color coordinate distance (CCD) is defined to describe the emission color and is calculated as follows

(1) $$\mathrm{CCD}=\frac{L_{\mathrm{hybrid}-\mathrm{GaN}}}{L_{\mathrm{Pe}-\mathrm{GaN}}}$$

where *L*_Pe − GaN_ is the distance between the color coordinates (CIE‐1931) of the PeLEDs and GaNLEDs and *L*_hybrid − GaN_ is the distance between the color coordinates of the Pe‐GaN tandem LEDs and GaNLEDs. A greater CCD indicates that the color coordinates of the tandem devices are close to those of green PeLEDs; conversely, a smaller CCD suggests that the emission is close to that of blue GaNLEDs. The CCD tuning range of tandem devices with different fixed *V*_GaN_ values is displayed in **Figure** **2**a, corresponding to a linear color coordinate change due to the stable emission spectra of both units (Figure S3, Supporting Information).

With a fixed *V*_GaN_ value, the upper limit of the CCD depends on the ratio between the luminance of the GaNLED and maximum luminance of the PeLED units. It is clear that such a ratio decreases with an increase in *V*_GaN_, leading to a blue shift of the upper limit of the CCD (i.e., a reduced maximum CCD value), as indicated in Figure 2a. The lower limit of the CCD depends on the combination of the EL of the GaNLED and photoluminescence (PL) of the PeLED (the EPCE state illustrated in Figure 2b), it indicates a green shift (i.e., an increased minimum CCD value) with increasing *V*_GaN_ value which makes different lower limits for CCD, these results are attributed to the enhanced PL green intensity of the PeLED unit. Fortunately, the lower limit stops the green shift when the *V*_GaN_ value is further increased (Figure S4, Supporting Information), which maintains the emission in the pure blue region (minimum CCD ≤ 0.05). To further investigate the PL effect of PeLED on the CCD tuning range, the luminance of the GaNLED at different current densities with and without the PeLED is displayed in Figure 2c. As the current density of the GaNLED increases, the luminance of the GaNLED without the PeLED (*L*_w/o‐Pe_) continues to increase, whereas that of the PeLED (*L*_w‐Pe_) demonstrates a clear roll‐off. Furthermore, *L*_w‐Pe_ is divided into the luminance from the green PL of the PeLED (*L*_G_) and that from the blue EL of the GaNLED (*L*_B_). We found that *L*_G_ exhibits the same trend as *L*_w‐Pe_, demonstrating that the roll‐off in *L*_w‐Pe_ originates from the degraded PL intensity of the perovskite layer, which could be attributed to trap state formation by ion exchange and crystal distortion of the perovskite under ultra‐strong short‐wavelength excitation.^[^ ^35^, ^36^ ^]^

Consequently, as indicated in Figure 2d, a wide CCD tuning range is realized by regulating the luminance of both units in the DC mode. In such a Pe‐GaN tandem LED, for example, the pure green color (CCD = 1) is obtained by solely driving the PeLED unit, and its luminance depends on the EL performance of the PeLED unit; pure blue (CCD = 0.01–0.048) is obtained by solely driving the GaNLED unit, and its luminance is dependent on the EL and PL performance of the GaNLED and PeLED units, respectively; the transition color from blue to green (CCD = 0.048–1) is obtained by simultaneously driving the GaNLED and PeLED units, and its luminance is comprehensively dependent on the EL performance of both units, as well as the PL performance of the PeLED unit. Video S1, Supporting Information is provided to demonstrate the color tunability of the Pe‐GaN tandem LED. Hence, the color gamut of the proposed Pe‐GaN tandem LED can achieve up to 104% NTSC with the maximum adjustable luminance when combined with a commonly used red emitter such as CsPbI_3_ PeLED.^[^ ^37^ ^]^

### 2.3 Luminance Tunability and EPCE Effect under DC Mode

**Figure 3:**
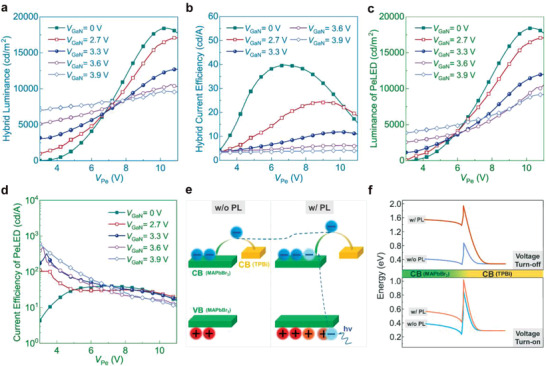
Luminance tunability of Pe‐GaN tandem LEDs under DC mode. a) Hybrid luminance. b) Hybrid CE. c) Luminance of PeLED unit. d) CE of PeLED unit. e) Illustration of suppressed current injection by PL process. The energy level is placed aligned to show the increased barrier. f) Finite element simulation of the energy level in the conduction band at the MAPbBr_3_/TPBi interface without and with applied voltage. All tandem devices were measured by varying the scanning voltage of the GaNLED and PeLED units.

The luminance tunability of Pe‐GaN tandem LEDs under DC mode was investigated with different emission colors by changing *V*_GaN_ and *V*_Pe_. **Figure** **3**a displays the hybrid luminance of Pe‐GaN tandem LEDs with different *V*_GaN_ and *V*_Pe_ values. When solely driving the PeLED (*V*_GaN_ = 0 V) to achieve a pure green color, a maximum hybrid luminance (HL_max_) of 18 482 cd m^−2^ is achieved at a *V*_Pe_ of 10.4 V. When driving both units to achieve a transition color, the EL (PeLED/GaNLED) and PL (PeLED) processes together establish the EPCE state in the tandem device, which causes a gradual reduction of the HL_max_ of Pe‐GaN tandem devices with increasing *V*_GaN_. This phenomenon indicates that the enhanced blue luminance of the GaNLED unit negatively influences the luminance of the PeLED, leading to a reduction in HL_max_.

Similarly, Figure 3b indicates that the greater luminance of the GaNLED leads to a lower hybrid CE of the Pe‐GaN tandem LED. For example, when only driving the PeLED unit, the maximum CE and EQE are 39.7 cd A^−1^ and 11.4%, respectively, whereas they decrease to 24.4 cd A^−1^ and 7%, respectively, when simultaneously driving the GaNLED unit with a *V*_GaN_ of 2.7 V. One possible reason for the degraded HL_max_ and CE is that the radiative recombination and current injection are suppressed by the EPCE effect, which has rarely been considered in previous studies of perovskite‐based multicolor EL devices with only one recombination center.

To further investigate the effect of EPCE, the luminance of the PeLED units with different *V*_GaN_ values is displayed in Figure 3c. The initial green luminance around the turn‐on voltage is enhanced with increasing *V*_GaN_ (e.g., achieving 4867 cd m^−2^ at a *V*_GaN_ of 3.9 V). These results originate from the dominant PL process of the PeLED at low *V*_Pe_. As the *V*_Pe_ increases gradually, the greater *V*_GaN_ results in a decrease in the maximum green luminance, which significantly decreases from 18 429 cd m^−2^ (*V*_GaN_ = 0 V) to 9650 cd m^−2^ (*V*_GaN_ = 3.9 V). Therefore, the CE of the PeLED unit achieves hundreds of cd A^−1^ at low *V*_Pe_ owing to the domination of the PL process, and continuously decreases with increasing *V*_Pe_ owing to the gradual transition to the domination of the EL process, as indicated in Figure 3d. A reduction in the overall CE of the PeLED unit is observed with a greater *V*_GaN_, demonstrating that a stronger blue light excitation results in a less efficient PeLED. These results indicate that more non‐radiative recombination occurs in the PeLED unit with blue light illuminance. The surface temperature of the GaNLED units (<35 °C) is considerably less than the annealing temperature of the perovskite layer (Figure S5, Supporting Information), and has a negligible influence on the degradation of the PeLEDs. Such increased non‐radiative recombination is likely attributable to the enhanced Auger recombination dominated at an ultra‐high charge carrier density,^[^ ^38^ ^]^ with extra‐photoexcitation in our case of EPCE. To support this notion, the blue light absorption *a*_B_ and green light conversion efficiency GLCE of the PeLED unit are defined as follows

(2) $$a_{B}=\frac{L_{w/o-\mathrm{Pe}}-L_{B}}{L_{w/o-\mathrm{Pe}}}$$

(3) $$\mathrm{GLCE}=\frac{L_{G}}{L_{w/o-\mathrm{Pe}}-L_{w-\mathrm{Pe}}}$$

where *L*_w‐Pe_, *L*_w/o‐Pe_, *L*_B_, and *L*_G_ are obtained from Figure 2c. In the case of PeLED units with a different *V*_GaN_, we found that the *a*_B_ increased whereas the GLCE continued to decrease (Figure S6, Supporting Information). These results indicate that more blue light is absorbed by the PeLED unit with increasing *V*_GaN_, resulting in more photoexcited charge carriers. Conversely, the GLCE continues to decrease with increasing *V*_GaN_, indicating that the non‐radiative recombination gradually dominates in PeLEDs. This implies that the increased photoexcited charge carriers from the blue light of the GaNLEDs are sufficient for Auger recombination to replace the radiative bimolecular recombination.^[^ ^39^ ^]^ As the defects in perovskite layer also influence the amount of charge carriers,^[^ ^40^ ^]^ the EPCE effect in tandem devices can be probably suppressed by modifying the perovskite structure in future. Additionally, it is also an alternative to avoid the blue light excitation to suppress the EPCE effect, such as integrating waveguiding structures.

Furthermore, we also studied the effect of EPCE on charge injection in PeLEDs. Figure S7, Supporting Information indicates that the PeLED unit exhibits a decreased current density with increasing *V*_GaN_; the current density of the PeLED unit is reduced from 113.4 to 85.2 mA cm^−2^ (*V*_Pe_ = 11 V) when increasing *V*_GaN_ from 0 to 3.9 V. These results can be further confirmed by the change in the current density of a PeLED unit with alternating ON/OFF GaNLEDs (Figure S8, Supporting Information). These results suggest that the charge injection of the PeLED unit is suppressed under blue light excitation. Such a photoexcitation process dramatically increases the electron density in the conduction band (and hole density in the valence band), forming a high‐level filling state that suppresses the electrical injection,^[^ ^41^ ^]^ as indicated in Figure 3e. The 1D device model was built in the COMSOL semiconductor module^[^ ^42^ ^]^ to provide an in‐depth understanding, and finite element simulation was conducted to obtain the energy level in the conduction band at the MAPbBr_3_/TPBi interface with and without optical transition, as indicated in Figure 3f (detailed setups can be found in Table S1, Supporting Information). Herein, the optical transition model is utilized to generate photo‐induced carriers for simulating the PL process with blue light illumination, whereas optical reabsorption and re‐emission in the perovskite layer^[^ ^43^ ^]^ is not considered. The results confirm that the injection barrier of the perovskite layer is increased in the case of photo‐induced carriers during the PL process, suppressing the carrier injection to the perovskite layer, and thereby reducing the current density and green luminance of the PeLED units. In other words, the EPCE effect not only strengthens the non‐radiative recombination of the perovskite, but also sets a barrier for charge injection, which is harmful to the overall performance of both the PeLED unit and Pe‐GaN tandem LED.

### 2.4 Luminance and Color Tunability under PWM Mode

**Figure 4:**
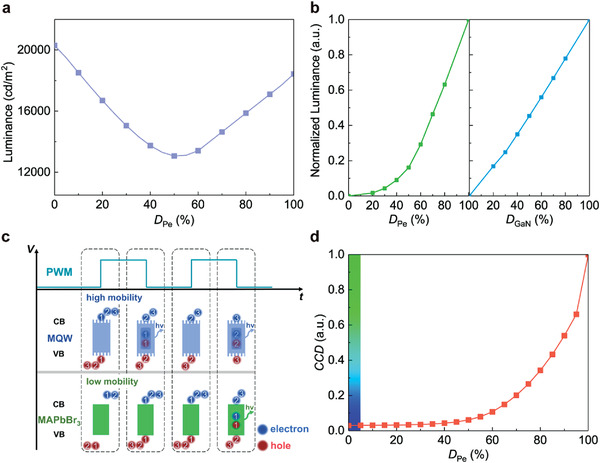
Luminance and color tunability of Pe‐GaN tandem LEDs under PWM mode. a) Hybrid luminance with different duty ratios of PeLED (*D*_Pe_). b) Normalized luminance of PeLED units (left) and GaNLED units (right) with different duty ratios. c) Illustration of carrier transport influenced by mismatched carrier mobility under PWM mode. d) CCD values with different *D*_Pe_ values. All tandem devices were measured with fixed maximum luminance of both units under DC mode. The driving frequency is 50 Hz.

Considering that the EPCE effect degrades the device performance when simultaneously driving GaNLED and PeLED units, herein, we propose a facile and effective strategy to suppress the EPCE effect for Pe‐GaN tandem LEDs, that is, the PWM mode that drives each unit alternately via modulated square‐wave voltages is introduced to avoid the EPCE effect. In our work, the luminance and emission color of Pe‐GaN tandem LEDs under PWM mode were precisely modulated by fixing the voltage amplitude of both units (10.4 V for PeLED and 6 V for GaNLED, corresponding to their maximum luminance under DC mode) and by adjusting the duty ratio, as indicated in Figure S9, Supporting Information. The tandem device emits a blue color when the duty ratio of PeLED units (*D*_Pe_) is zero, whereas its emission color shifts to cyan and finally to pure green with increasing *D*_Pe_. Notably, the driving frequency is taken as 50 Hz, which allows the human eye to perceive the hybrid emission from two subpixels.^[^ ^25^ ^]^ **Figure** **4**a displays the hybrid luminance with different *D*_Pe_ values in PWM mode. When the *D*_Pe_ is zero, only the GaNLED unit is turned on, with an HL_max_ of 20 298 cd m^−2^. However, the HL_max_ decreases to a minimum of 13 061 cd m^−2^ with increasing *D*_Pe_. These results demonstrate that the enhanced luminance of the PeLED units with increasing *D*_Pe_ is insufficient to compensate for the reduced luminance of the GaNLED units, although the maximum luminance of both units is similar (≈20 000 cd m^−2^). As the *D*_Pe_ further increases, the EL intensity of the PeLED unit dominates the hybrid luminance, which gradually increases HL_max_ to 18 482 cd m^−2^ (the maximum luminance of the PeLED unit).

To better understand this phenomenon, the normalized luminance of the PeLED and GaNLED units with different duty ratios is displayed in Figure 4b. When the *D*_Pe_ is less than 50%, the PeLED unit demonstrates a nonlinear increase in luminance with increasing *D*_Pe_, which can be attributed to the relatively low carrier mobility of transport layers in the PeLED units^[^ ^44^ ^]^ which limits the response speed of the device under a short applied voltage. Conversely, the GaNLED unit has a linear relationship between its luminance and duty ratio (*D*_GaN_), which can be attributed to the high carrier mobility of the transport layers,^[^ ^45^ ^]^ as illustrated in Figure 4c. Fortunately, in a large *D*_Pe_ region (>50%), the luminance of the PeLED increases linearly, and hence successfully compensates the hybrid luminance. Therefore, we can conclude that the deep “valley” in Figure 4a results from the slow response speed of the PeLED. The CCD tuning range of the tandem devices under the PWM mode is also indicated in Figure 4d, reflecting the excellent color tuning ability and high color gamut (Figure S10, Supporting Information), as under DC mode.

**Figure 5:**
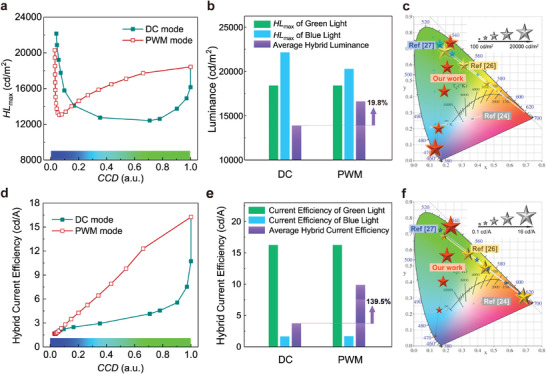
Independent tunability of luminance and color for Pe‐GaN tandem LEDs at maximum hybrid luminance (HL_max_) state. a) HL_max_ at arbitrary CCD values from blue to green color. b) HL_max_ comparison among different diving modes. c) Maximum hybrid luminance of reported perovskite‐based multi‐color LEDs. d) Hybrid CE at arbitrary CCD values from blue to green color. e) Hybrid CE comparison among different diving modes. f) Hybrid CE of reported perovskite‐based multi‐color LEDs. The hybrid CE corresponds to the HL_max_.

In practical display applications, the luminance and CCD values should be independently adjustable to realize excellent tunability for advanced display standards such as high‐dynamic‐range displays. In general, a greater maximum luminance at an arbitrary emission color demonstrates a greater overall brightness and contrast ratio for a full‐color display system.^[^ ^46^ ^]^ To confirm the excellent tunability of our tandem devices, we characterized their HL_max_ at different CCD values under PWM and DC modes, as displayed in **Figure** **5**a. Herein, the HL_max_ in PWM mode is achieved using the maximum luminance of each unit, as discussed above, whereas that under the DC mode, it is achieved by scanning the *V*_Pe_ or *V*_GaN_ with a fixed maximum luminance of GaNLED units (21 657 cd m^−2^) or PeLED units (18 429 cd m^−2^). Regarding Pe‐GaN tandem LEDs under DC mode, the theoretical HL_max_ for transition colors should be the superposition of both units (i.e., ≥40 000 cd m^−2^), whereas a severe reduction (actual HL_max_ of 12 414 cd m^−2^) due to the existence of EPCE can be observed. Because the PWM mode intrinsically eliminates the EPCE effect, it indicates a considerably narrower valley for the HL_max_ curves compared with the DC mode. To simplify the discussion, the average HL_max_ (i.e., the integration of HL_max_ with the CCD values) is defined to evaluate the overall HL_max_ of tandem devices under arbitrary colors. Accordingly, Figure 5b summarizes the HL_max_ of tandem devices under pure blue and green colors, and the average HL_max_ under arbitrary colors from blue to green. The HL_max_ for green colors is similar under both PWM and DC modes, whereas the HL_max_ for pure blue under the PWM mode is marginally less than that under the DC mode. This is because the PWM mode is without extra luminance from the PL process, and has a nonlinearly increased luminance of PeLED units with greater *D*_Pe_, especially in the small CCD range. However, the PWM mode continues to demonstrate a high average HL_max_ of 16 631 cd m^−2^, as it avoids the EPCE effect, which is an increase of 19.8% compared with the DC mode. Our devices maintain a large hybrid luminance (>14 000 cd m^−2^) for arbitrary tunable color, achieving the record tunable luminance in reported perovskite‐based multi‐color LEDs as shown in Figure 5c. Although such a hybrid luminance is still lower than that of other tandem devices, that is, tandem quantum dot LEDs,^[^ ^23^ ^]^ our proposed Pe‐GaN tandem LEDs successfully realize a record luminance tuning range under arbitrary color from the blue to green region for the first time in the class of perovskite‐based multicolor EL devices.^[^ ^24^, ^26^, ^27^ ^]^

In addition to the excellent luminance tunability, powerful Pe‐GaN tandem LEDs with significantly improved efficiency were also achieved by introducing the PWM mode. The hybrid CE under different CCD values is displayed in Figure 5d, which corresponds to HL_max_, as discussed above. It can be expected that the PWM mode, which avoids the EPCE effect and leads to a reduced hybrid current density (Figure S11, Supporting Information), indicates an overall greater hybrid CE compared with the DC mode. Similarly, based on the total luminance and current of the tandem devices, the average hybrid CE corresponding to HL_max_ was also calculated, as displayed in Figure 5e. The PWM mode demonstrates an average hybrid CE of 9.1 cd A^−1^, which is 139.5% greater than that of the DC mode. Our devices also achieve one of the highest hybrid CE in reported perovskite‐based multi‐color LEDs as shown in Figure 5f. The hybrid EQE, corresponding to HL_max_ of Pe‐GaN tandem LEDs under the PWM mode was also measured as shown in Figure S12, Supporting Information. The excellent luminance‐ and color‐independent tunability reported herein can be the starting point for multi‐color PeLEDs. The integration of GaNLEDs with PeLEDs also proves the success of introducing mature semiconductor technologies in perovskite‐based EL devices, significantly facilitating their commercialization of advanced display applications with ultra‐high resolution.

## 3 Conclusions

In this study, Pe‐GaN tandem LEDs were developed to achieve excellent luminance‐ and color‐independent tunability. The green emitter MAPbBr_3_ PeLED was deposited on a GaNLED substrate, serving as the blue emitter, where an ITO ICE was utilized as the co‐anode to realize parallel connection with independently tunable luminance and color. We found that the complicated EL (GaNLED/PeLED) and PL (PeLED) processes simultaneously occur in tandem devices under DC mode, resulting in the EPCE effect that suppresses the radiative recombination and current injection of the PeLED. With the serious EPCE‐induced energy loss, the hybrid luminance and CE were significantly degraded under the DC mode compared with those when driven separately. Hence, the PWM mode was introduced to the Pe‐GaN tandem LEDs with a negligible EPCE effect. Although they demonstrated marginal degraded hybrid luminance with a bluish transition color owing to the slow response speed of the PeLEDs, the average HL_max_ (16 631 cd m^−2^) and hybrid CE (9.1 cd A^−1^) were 19.8% and 139.5%, respectively, greater than the values under DC mode. Accordingly, a record wide‐range tunable luminance and significantly improved efficiency were achieved for Pe‐GaN tandem LEDs at an arbitrary color from blue to green. We believe that such excellent luminance‐ and color‐independent tunability can be a starting point for perovskite‐based multi‐color EL devices, enabling the combination with matured semiconductor technologies to facilitate their commercialization in advanced display applications with ultra‐high resolution.

## 4 Experimental Section

### Materials

PEDOT:PSS (4083) was purchased from Heraeus Electronic Materials Division. Methylammonium bromide (MABr, > 99.99%), lead bromide (PbBr_2_, > 99.99%), and TPBi (> 99%) were purchased from Xi'an Polymer Light Technology Corp. DMF, DMSO, and chlorobenzene (HPLC, > 99.9%) were purchased from Aladdin. Blue GaN wafers were purchased from Foshan National Semiconductor Co. Ltd., All chemicals were used as received without further purification.

### GaNLED Substrate Fabrication

The received blue GaN wafer was prepared based on the standard epitaxial process of metal organic chemical vapor deposition, which consisted of PSS (600 µm), u‐GaN (3 µm), Si‐doped n‐GaN (2.5 µm), InGaN/GaN (120 nm), InGaN/GaN quantum well (30 nm), p‐AlGaN/GaN (48 nm), and Mg‐doped p‐GaN (110 nm) from bottom to top. The GaN wafer was locally etched from the p‐GaN surface to n‐GaN using inductively coupled plasma etching (Cl_2_/BCl_3_ as the etching gas) with an etching depth of ≈1100 nm. A SiO_2_ passivation layer with a thickness of 200 nm was patterned on the n‐GaN surface by chemical vapor deposition and wet etching. An ITO layer with a thickness of 230 nm was deposited on the surface of p‐GaN to prepare the anode using magnetron sputtering with a sheet resistance of approximately 20 Ω□^−1^ after the annealing treatment. The ITO layer was patterned using a shadow mask. Then, Cr (2.5 nm), Al (100 nm), Ti (105 nm), Pt (850 nm), and Au (1500 nm) were subsequently evaporated within the region of the n‐GaN layer without SiO_2_ passivation for cathode preparation. After electrode deposition, the GaN wafer was thinned to 175 µm by grinding the PSS, which was finally cut into square GaNLED substrates with a size of 20 × 20 mm for subsequent PeLED fabrication.

### Pe‐GaN Tandem LED Fabrication

The Pe‐GaN tandem LED was fabricated by depositing PeLEDs on a GaNLED substrate. First, the GaNLED substrate was cleaned successively using toluene, isopropanol, and ethanol in a sonic bath, followed by drying in a vacuum oven. After 4 min of oxygen plasma treatment of the substrate, PEDOT:PSS was spin‐coated on the substrate at 4000 rpm, followed by baking at 120 °C for 15 min under ambient conditions. The GaNLED substrate was then transferred into a vacuum chamber inside an Ar‐filled glovebox (H_2_O and O_2_ ≤ 0.1 ppm), followed by thermal evaporation of LiF (1.5 nm) at an evaporation rate of 0.1 Å s^−1^. The perovskite precursor solution (20 wt%) was prepared by dissolving MABr and PbBr_2_ in a molar ratio of 1.1:1 in a DMF/DMSO mixed solution (V_DMF_: V_DMSO_ = 7:3). To form the perovskite layer, 75 µL of the precursor solution was spin‐coated onto the GaNLED substrate at a speed of 3000 rpm for 60 s. After 15 s of spin‐coating, 50 µL of chlorobenzene was quickly dropped onto the center of the substrates, followed by baking at 60 °C for 10 min. Finally, TPBi (60 nm), LiF (1.5 nm), and Al (100 nm) were successively evaporated on the perovskite layer under a high vacuum of 4 × 10^−5^ Pa with evaporation rates of 1, 0.1, and 3 Å s^−1^, respectively.

### Characterization

The film thickness was measured using an SQC‐310C thin‐film deposition system (Inficon). The transmittance and haze spectra were recorded using a UV–vis spectrometer (Shimadzu) in the wavelength range of 300–800 nm at 1‐nm intervals. Scanning electron microscope images were obtained using a field‐emission scanning electron microscope (Zeiss, Merlin). The impedance measurements were performed using a DH7000 electrochemical workstation. The current density, bias, and luminance measurements were conducted using a Keithley 2450 source meter and a Konica Minolta Chroma Meter CS‐200. The EL spectra were recorded using a PhotoResearch PR‐705 photometer. The EQE was calculated from the luminance, current density, and EL spectra data, assuming a Lambertian distribution.

## Conflict of Interest

The authors declare no conflict of interest.

## Author Contributions

Z.‐T.L., J.‐S.L., and H.‐L.Y., conceived and designed the experiments. K.C., H.‐W.Z., L.X., and B.‐H.Y., performed the device fabrication and measurements. H.‐W.Z., X.‐R.D., and J.‐S.L., performed the simulations. H.‐L.Y., H.‐C.K., J.‐Z.O., and Z.‐T.L., contributed significantly to analysis and manuscript preparation. J.‐S.L., H.‐W.Z., K.C., and Z.C., wrote the manuscript and drew the figures. All authors contributed to result discussions and manuscript revisions.

## Supporting information

Supporting Information

Supplemental Video 1

## Data Availability

The data that support the findings of this study are available from the corresponding author upon reasonable request.
