# Supplementary material for: Perovskite‐Gallium Nitride Tandem Light‐Emitting Diodes with Improved Luminance and Color Tunability
Source: Adv Sci (Weinh). 2022 May 21;9(22):2201844. doi: 10.1002/advs.202201844 (PMC9353454; doi:10.1002/advs.202201844)
Supplement: Supplementary file 1 — Supporting Information [file ADVS-9-2201844-s002.pdf]

Supporting Information

Perovskite-Gallium Nitride Tandem Light-Emitting Diodes with Improved Luminance and Color Tunability

Zong-Tao Li,[1] Hong-Wei Zhang,[1] Jia-Sheng Li,[1*] Kai Cao,[1] Ziming Chen,[7] Liang Xu,[8] Xin-Rui Ding,[1] Bin-Hai Yu,[1] Yong Tang,[1] Jian-Zhen Ou,[6] Hao-Chung Kuo[3], and Hin-Lap Yip[2, 4, 5]

[1]National & Local Joint Engineering Research Center of Semiconductor Display and Optical Communication Devices, South China University of Technology, Guangzhou 510641, China

[2]Department of Materials Science and Engineering, City University of Hong Kong, Kowloon, Hong Kong

[3]Department of Photonics and Institute of Electro-Optical Engineering College of Electrical and Computer Engineering, National Chiao Tung University, Hsinchu 30010, Taiwan, China

[4]School of Energy and Environment, City University of Hong Kong, Kowloon, Hong Kong

[5]Hong Kong Institute for Clean Energy, City University of Hong Kong, Kowloon, Hong Kong

[6]School of Engineering, RMIT University Melbourne, Victoria 3000, Australia

[7]Department of Chemistry, Imperial College London, London W12 0BZ, United Kingdom

[8]Foshan Nationstar Semiconductor Technology Co. Ltd., Foshan 528000, China

* jiasli@scut.edu.cn

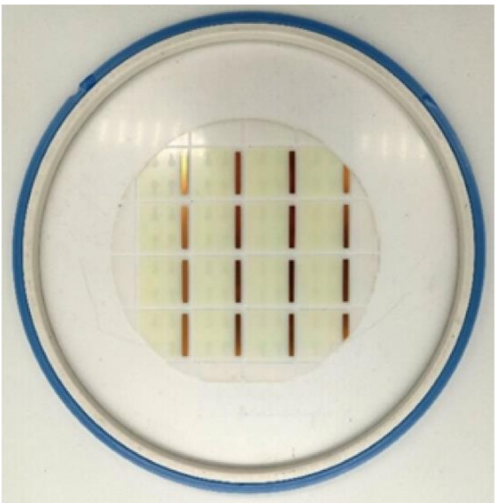

Figure S1. Image of GaNLEDs as the substrate for PeLED deposition.

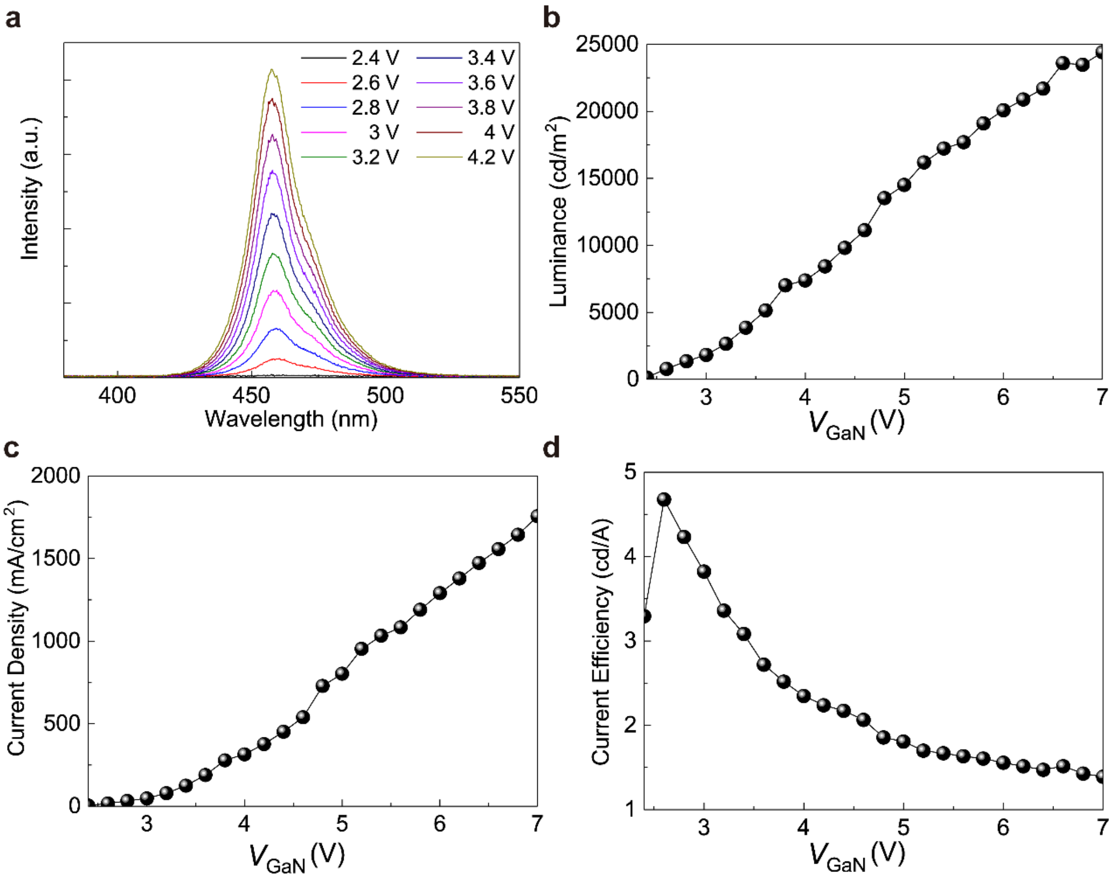

Figure S2. EL performance of GaNLEDs. (a) EL spectra. (b) Luminance. (c) Current density. (d) CE. The GaNLED is driven by scanning its voltage.

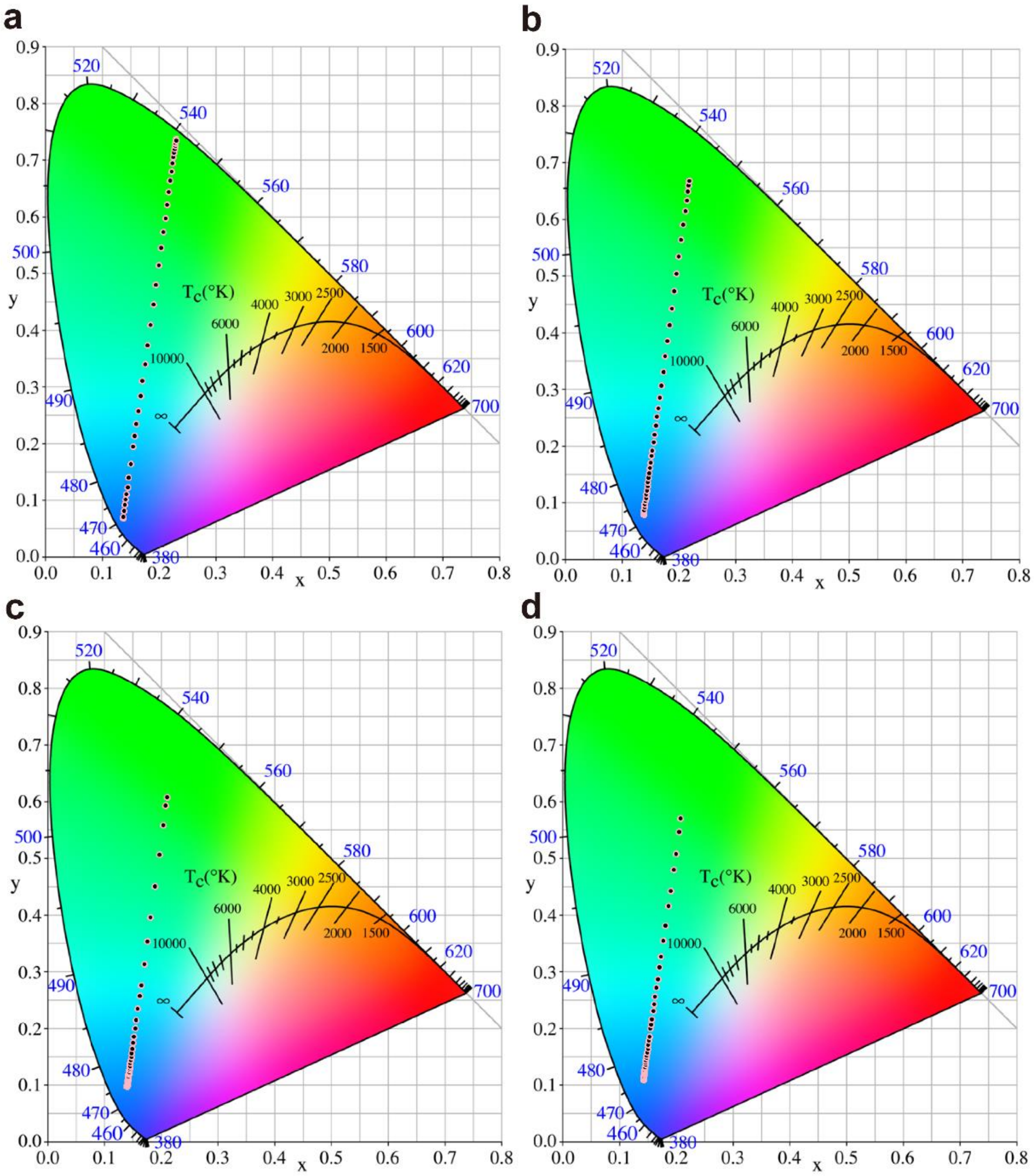

Figure S3. Color coordinates of Pe-GaN tandem LEDs under DC mode. (a)–(d) V_{GaN} values are 2.7, 3.3, 3.6, and 3.9 V, respectively.

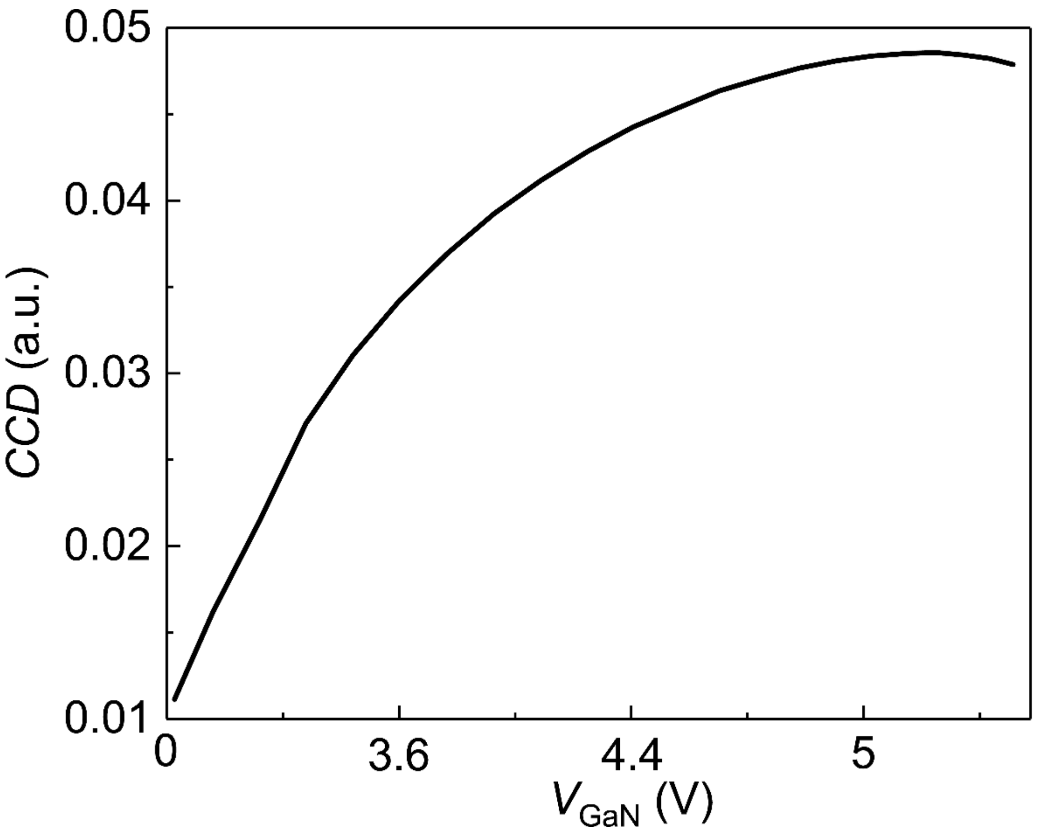

Figure S4. ***CCD*** **of Pe-GaN tandem LEDs with different** $\boldsymbol{V_{GaN}}$**.**

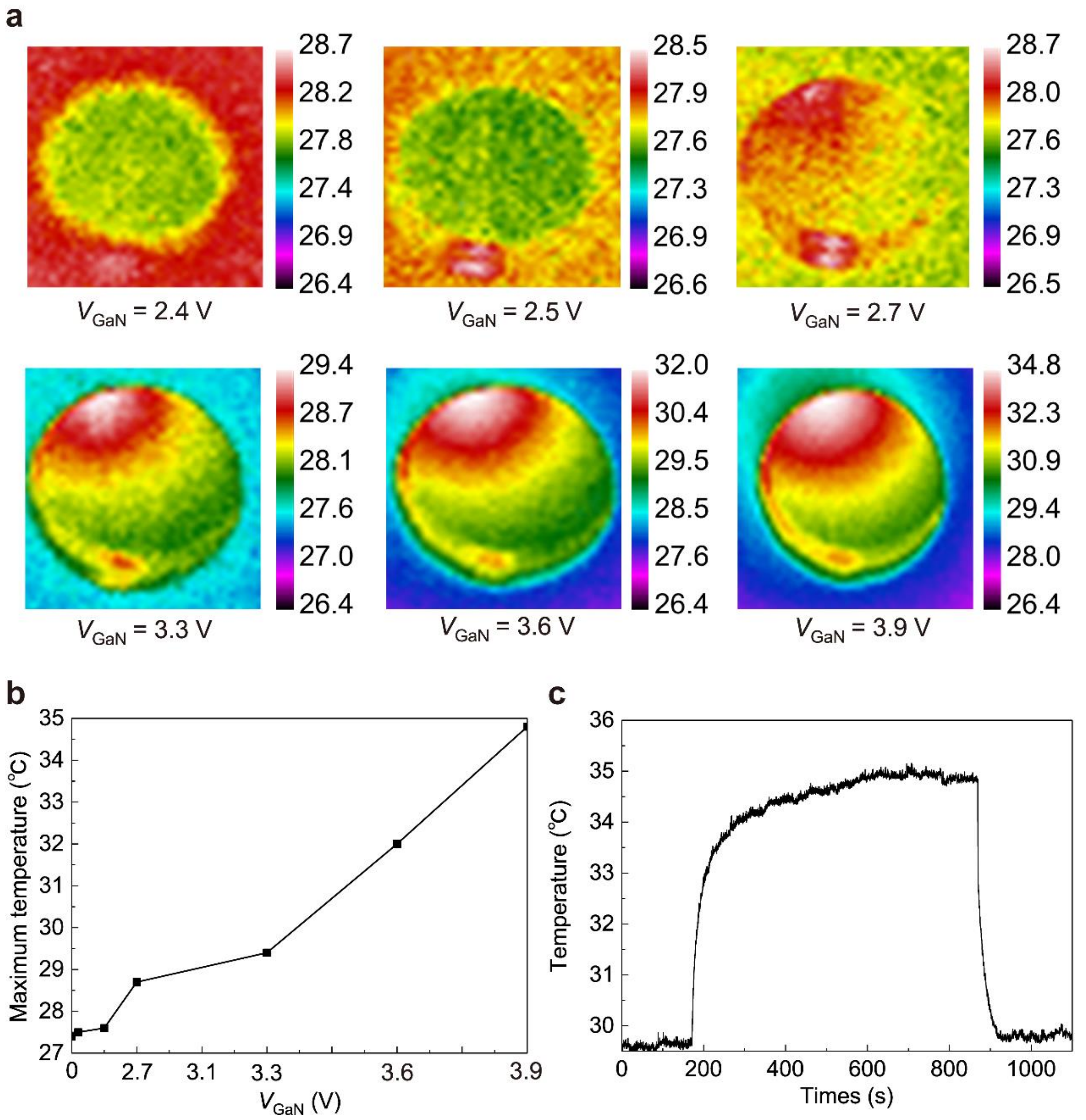

Figure S5. Thermal performance of GaNLEDs. (a) Surface temperature distribution with different V_{GaN}. (b) Maximum surface temperature with different V_{GaN}. (c) Heating and cooling curve at V_{GaN} of 3.9 V.

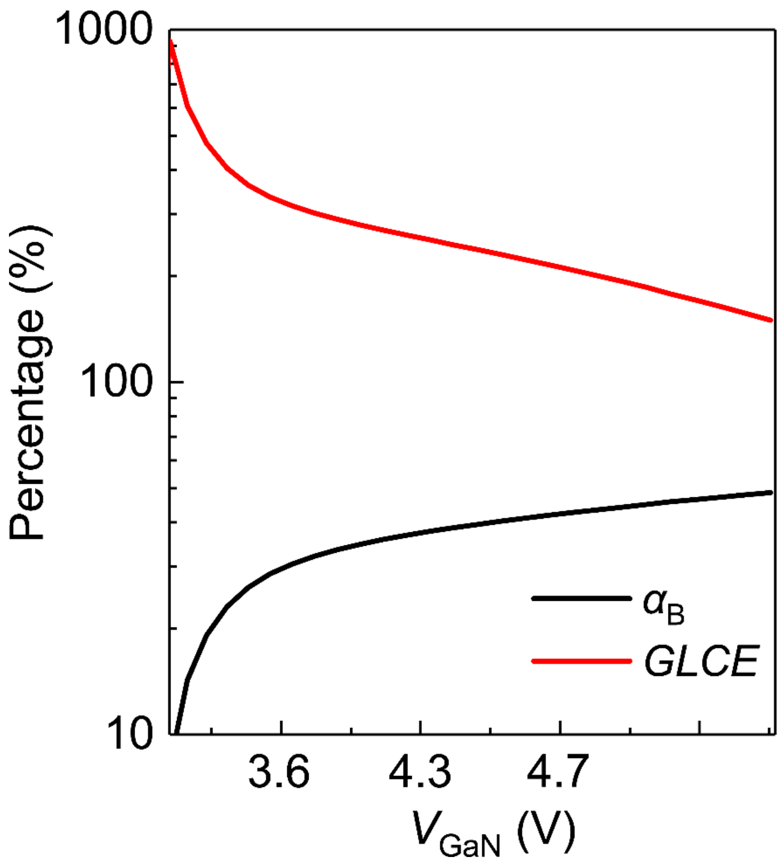

Figure S6. Blue light absorption rate α_B and *GLCE* of perovskite layers with different V_{GaN}.

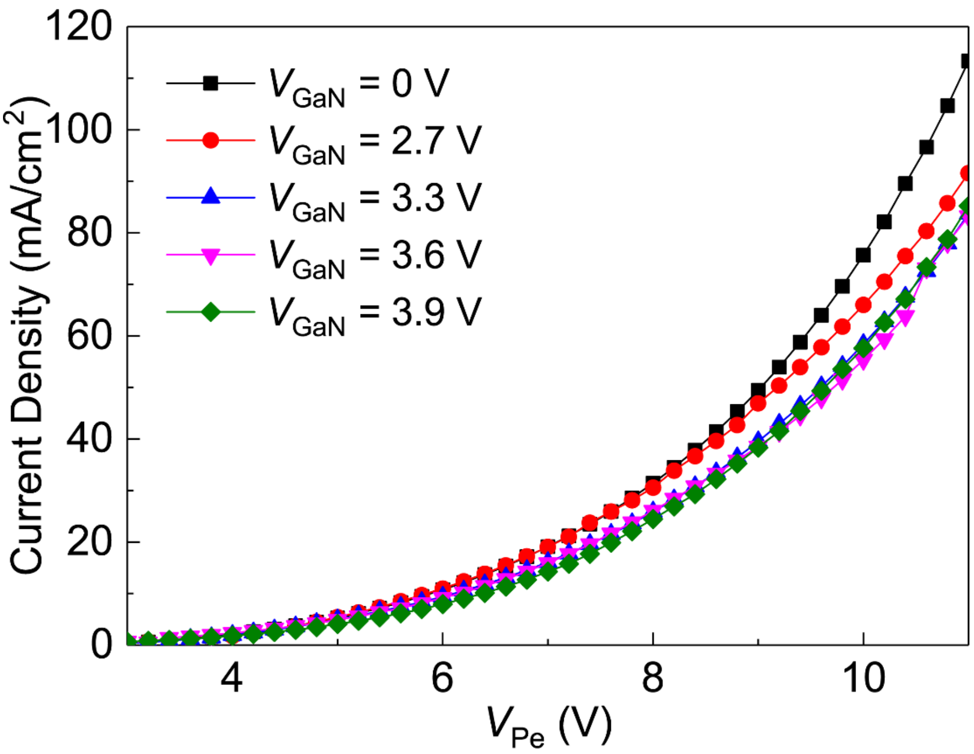

Figure S7. Current density of PeLED units in tandem devices under DC mode.

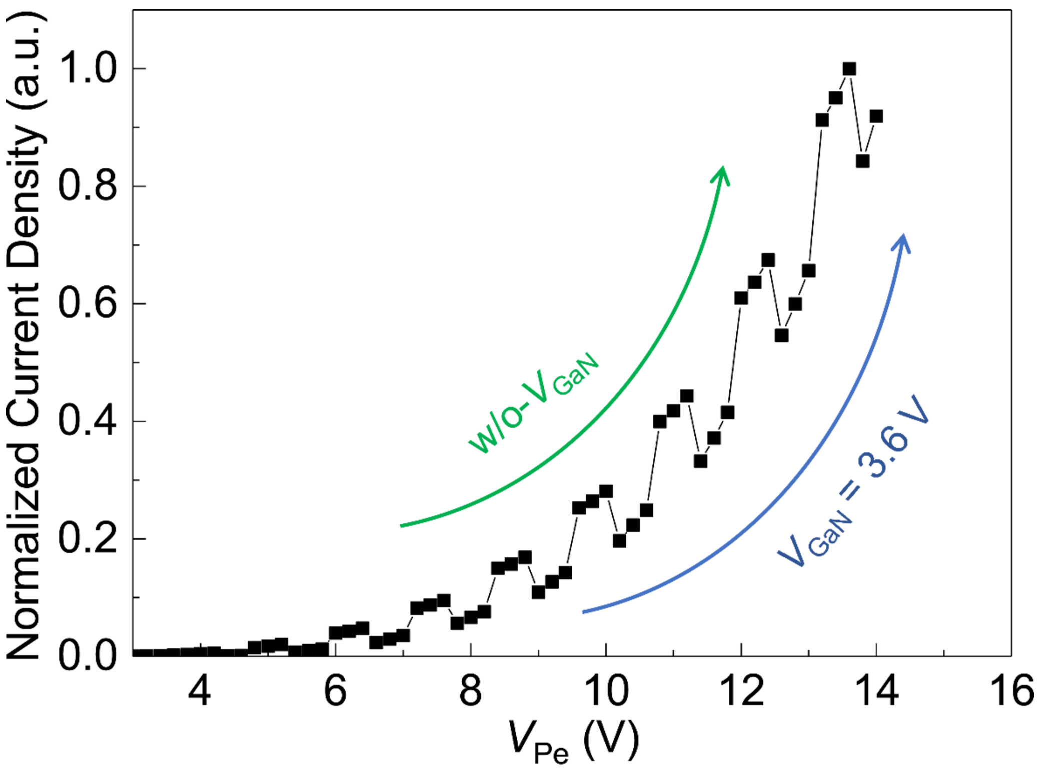

Figure S8. Normalized current density of PeLED unit with alternating ON/OFF GaNLED.

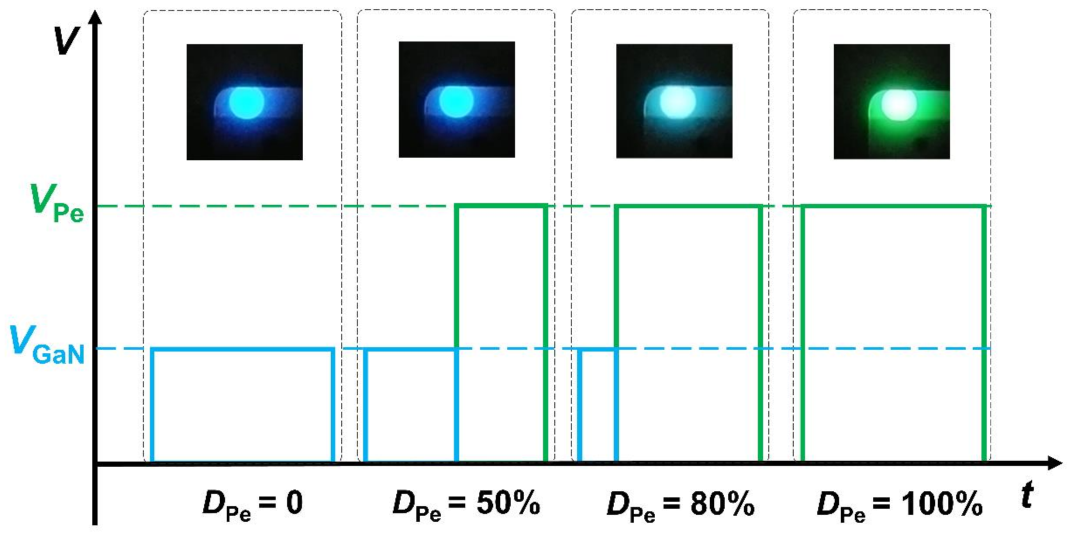

Figure S9. Illustration of Pe-GaN tandem LEDs under PWM mode using different duty ratios.

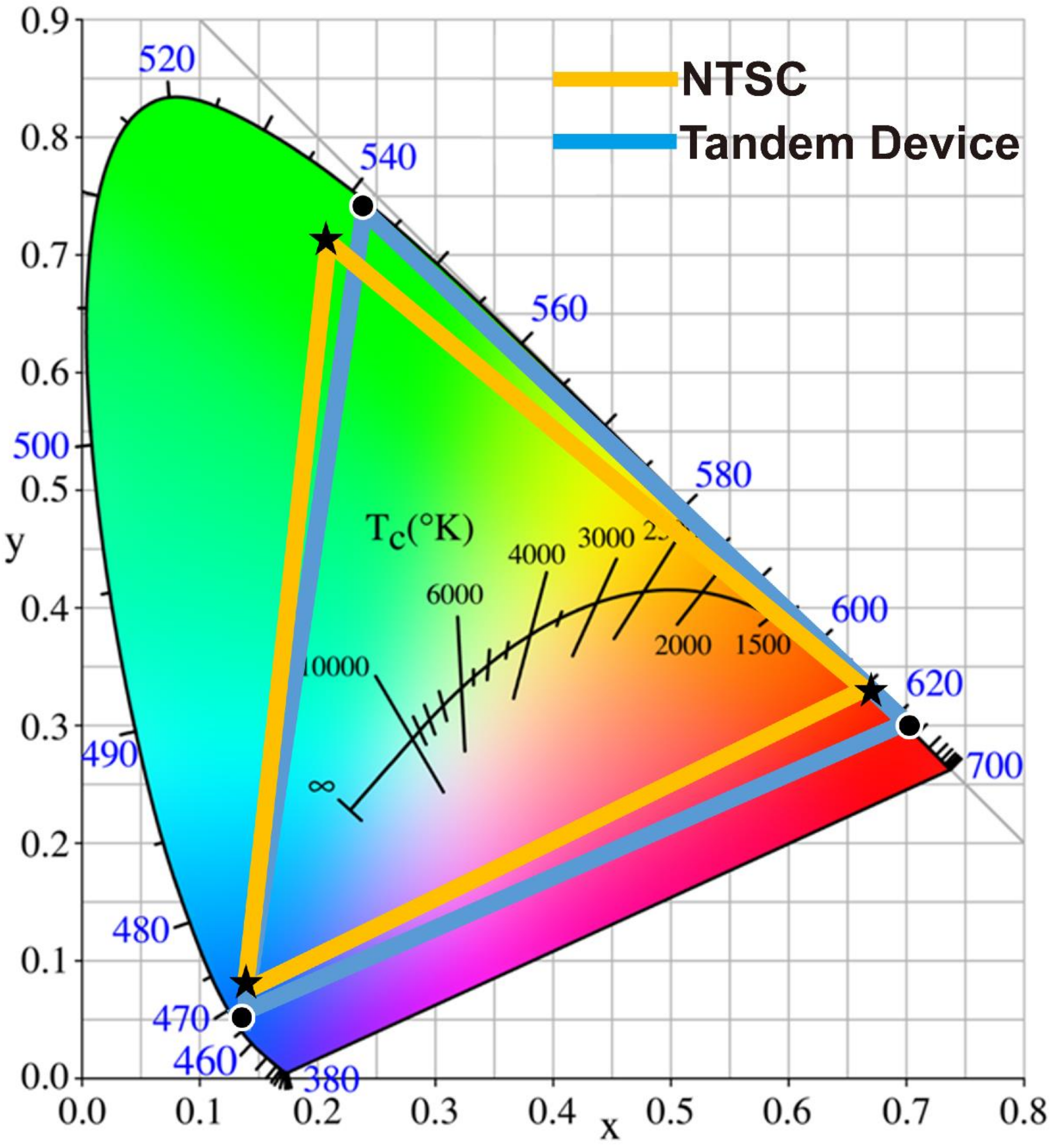

Figure S10. Color gamut of Pe-GaN tandem LEDs under PWM mode (f = 50 Hz) combined with a common red emitter.

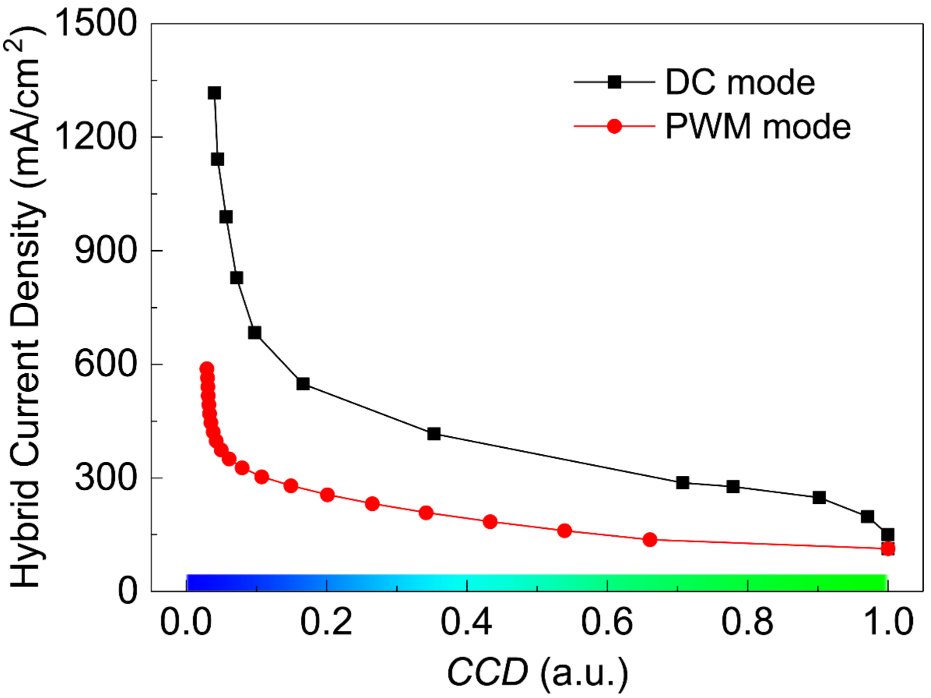

Figure S11. Hybrid current density of Pe-GaN tandem LEDs at arbitrary *CCD* values from blue to green.

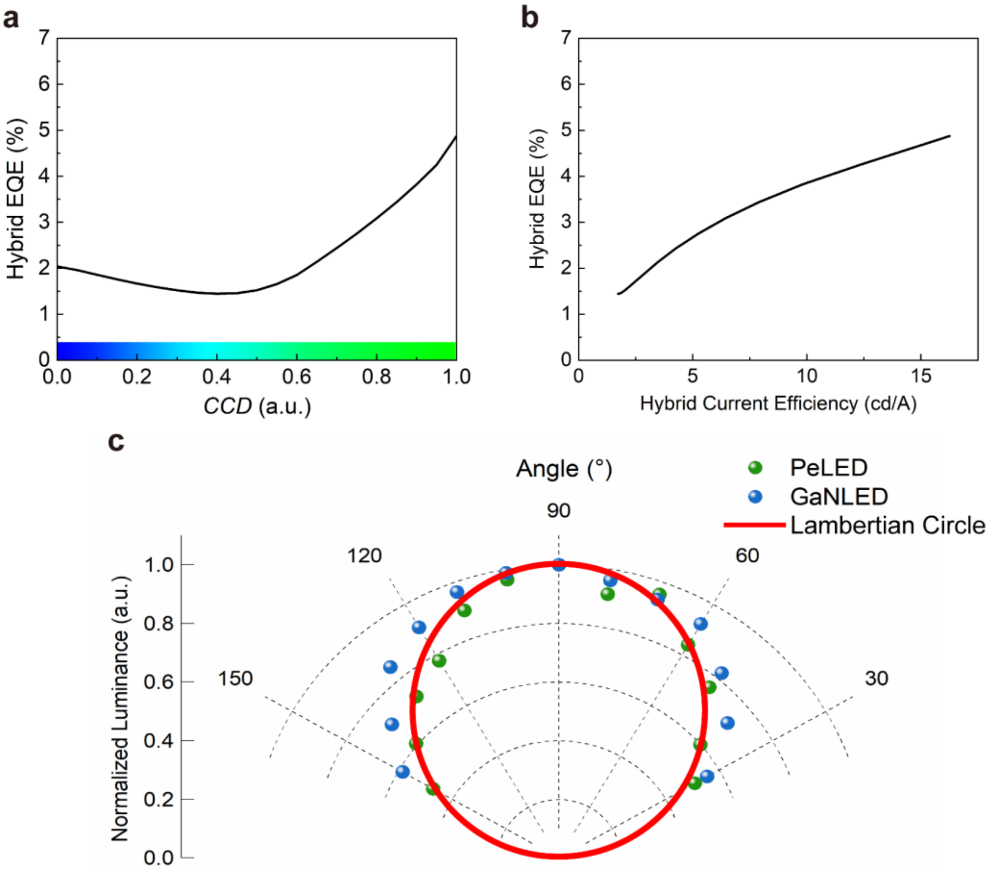

Figure S12. (a) Hybrid EQE of Pe-GaN tandem LEDs at arbitrary *CCD* values from blue to green. (b) Hybird EQE of Pe-GaN tandem LEDs versus Hybrid Current Efficiency. (c) Angle-dependent

normalized luminance of PeLED/GaNLED.

Table S1. Data used in COMSOL Semiconductor Module.

	HTL	LiF	Pero	ETH
T	293.15 [K]	293.15 [K]	293.15 [K]	293.15 [K]
th	40 [nm]	2 [nm]	500 [nm]	60 [nm]
ε_r	1	1	1	1
E_g	1 [V]	4.3 [V]	2.3 [V]	3.5 [V]
χ	4 [V]	1 [V]	3.4 [V]	2.7 [V]
N_v	1e20 [1/cm^3]	-	1e20 [1/cm^3]	1e20 [1/cm^3]
N_c	1e20 [1/cm^3]	-	1e20 [1/cm^3]	1e20 [1/cm^3]
μ_n	2e-6 [cm^2/V/s]	1e-9 [cm^2/V/s]	10 [cm^2/V/s]	1e-4 [cm^2/V/s]
μ_p	2e-4 [cm^2/V/s]	1e-7 [cm^2/V/s]	10 [cm^2/V/s]	6e-4 [cm^2/V/s]
τ_n	10 [μs]	10 [μs]	20 [μs]	50 [μs]
τ_p	10 [μs]	10 [μs]	20 [μs]	20 [μs]
τ_{spno}	-	-	30 [ns]	-
m_e*	-	-	0.2	-
m_h*	-	-	0.2	-
E_0	-	-	50 [V/m]	-
λ_0	-	-	455 [nm]	-
n	-	-	2.3	-
k	-	-	0.7	-
